# Opinions on Kampo and reasons for using it – results from a cross-sectional survey in three Japanese clinics

**DOI:** 10.1186/1472-6882-13-108

**Published:** 2013-05-16

**Authors:** Lydia Hottenbacher, Thorolf ER Weißhuhn, Kenji Watanabe, Takashi Seki, Julia Ostermann, Claudia M Witt

**Affiliations:** 1 Institute for Social Medicine, Epidemiology and Health Economics, Charité University Medical Center, Berlin D-10098, Germany; 2 Center for Kampo Medicine, Keio University School of Medicine, 35 Shinano-machi, Shinjuku-ku, Tokyo 160-8582, Japan; 3 Department of Geriatric Behavioral Neurology, Tohoku University Graduate School of Medicine, 1-1 Seiryo-machi, Aoba-ku, Sendai 980-8575, Japan; 4 University of Maryland School of Medicine, Center for Integrative Medicine, Baltimore, USA

**Keywords:** Kampo, Survey, Patient opinions

## Abstract

**Background:**

Traditional Japanese Medicine (Kampo) is often used in Japan, but very little data on its users are available. We investigated who uses Kampo, the reasons and opinions for its use.

**Methods:**

Questionnaire survey in three Japanese outpatient clinics offering Kampo in different settings: Kampo only, Kampo and traditional Chinese medicine, Kampo and Western medicine. Before seeing the doctor, patients were asked about socio-demographic data, medical history, experience with Kampo, general health-related opinions and behaviours, opinions about Western medicine and Kampo, and reasons for Kampo utilization. Descriptive statistics and predictors for Kampo use were calculated.

**Results:**

A total of 354 questionnaires were completed. Participants were 50.97 ± 15.60 (mean ± SD) years of age, 68% were female. Of all patients, 73% (n = 202) were using Kampo currently and 84% (297) had taken Kampo before. Questions on general health-related opinions and behaviour revealed a strong environmental awareness. The most frequent indications for earlier Kampo use were: common cold (36%), gastrointestinal complaints (30%), oversensitivity to cold (“Hi’e-sho”; 29%), stress/anxiety (21%), and shoulder stiffness (20%). Kampo users suffered more often from chronic illnesses (OR 2.88 [1.48-5.58]). Beliefs in underlying philosophy (Wu Xing (adjusted OR 3.08, [1.11-8.55]), Ying and Yang (OR 2.57 [1.15-5.73], a holistic way of seeing the patient (OR 2.17 [1.53-3.08]), and in Kampo efficacy (OR 2.62 [1.66-4.13]) were positively associated with Kampo use. So was, interestingly, conviction of the efficacy of Western medicine (OR 1.87 [1.28-2.74]). Half of the patients had a general preference for a combination of Kampo and Western treatment.

**Conclusions:**

Most patients visiting a clinic that also provided Kampo had previous experience with Kampo. Usage was associated with beliefs in philosophical Kampo concepts and its efficacy.

## Background

Complementary and alternative medicine (CAM) is increasingly used worldwide [1], especially in industrialized countries including Japan [2-4]. The CAM therapies most used in Japan in the general population during the last 12 months before a telephone survey were nutritional and tonic drinks as well as dietary supplements (43.1% each), Kampo (17.2% over-the-counter plus 10.0% prescribed), health-related devices (21.5%), massage or acupressure (14.8%), and aromatherapy (9.3%) [3]. Kampo (also: Kanpo, 漢方, or Kampo Igaku, 漢方医学) is a traditional medicine used almost solely by Japanese. Among those with diseases, it is the most popular CAM modality [5,6].

Although rooted in Chinese tradition, Kampo medicine is not the same as Chinese medicine (CM). CM emphasizes the traditional concepts of East Asian natural philosophy, such as Yin and Yang or the theory of the Five Elements. Japanese Kampo favours diagnostic methods that directly relate symptoms to therapy, bypassing speculative concepts. The vast array of crude drugs has been reduced in Kampo and also the quantity of each drug in the formulation is much lower. While Kampo maintains traditional prescriptions, CM also tends to create new drug combinations [7,8]. A Kampo diagnosis (shô, 証; a kind of constitutional state [9]) is based on history taking, inspection (including tongue), smell, listening to body sounds, and palpation of pulse and abdomen [10]. The treatment (hô, 方) usually consists of herbal preparations. Animal or mineral preparations, acupuncture, or moxibustion are rarely applied [11,12].

Kampo had been supplanted with the “medicine from the West” (seiyô igaku, 西洋医学), the Western biomedicine that is often referred to as “conventional” medicine in the Western world. Kampo was nearly nonexistent in the early 20^th^ century, but has seen a strong resurgence in popularity. Some examples shall illustrate this: Even before 1980 a colloquial term “kanpo boomu” [sic], “the Kampo boom”, existed [13]. In 2005, Kampo was practiced by 78% of the doctors in Kyoto and was taught in all Japanese medical schools [14]. The 2009 annual Japanese expenditure on Kampo products reached about 1 billion USD, excluding over-the-counter (OTC) sales [15]. A review of clinical research on Kampo in 2011 identified 135 studies, which indicates a growing research interest [7]. A corresponding methodology to satisfy both Kampo and Western diagnostic systems has been developed [7]. Because it has been included in, or subordinated to, the Western health care system [16], (about 90% of its costs are now reimbursed by the national healthcare system), it has been tentatively suggested that Kampo is no longer an “alternative” or “complementary” medical system [17]. Nowadays a prescription is seldom based on the traditional shô diagnosis alone but used as co-medication for a Western-diagnosed (ICD-10) disease [18].

Although one out of ten Japanese is prescribed Kampo [3], very little data on its use and users are available. A small number of surveys have been performed in Kampo outpatient clinics [6,19-22]. Earlier works lacked usage data [13,23,24], while in more recent surveys on the use of CAM Kampo was only listed as one of several treatment modalities but was not investigated in detail [3-5,25-28]. We therefore performed a survey to gain more insights into Kampo use. Especially the reasons why patients use it, what they think about it, and how they see it in relation to Western medicine were investigated in patients of three clinics that offer Kampo treatment. The term “Kampo” in this paper will include both the actual use of Kampo therapies as well as the decision for it (covering the whole range from a full shô diagnosis by an expert to an advertisement-induced layman’s decision).

## Methods

We performed a multicenter cross-sectional survey between 1^st^ September and 30^th^ October 2008 in three Japanese clinics that offer Kampo treatment. In this paper they will be labelled as follows:

•“*Kampo”:* Outpatient clinic at a large private university hospital in Tokyo. Only Kampo is practiced (classically, i.e., based on a shô diagnosis).

•“*Kampo/CM”:* Public university hospital in Sendai. Kampo is used primarily; in cases of insufficient improvement acupuncture and moxibustion from Chinese Medicine (CM) are used. Patients are almost always treated as outpatients, but 20 beds (out of 1000) are available for Kampo patients.

•“*Integrative”:* The clinic of an internist in own practice in Sendai who practices Western medicine, Kampo among various other CAM therapies, as well as counselling.

The original survey included a fourth clinic that provided only Western medicine. This data has been presented in LH’s thesis [29]. The present paper focuses on understanding Kampo users and includes only the Kampo providing clinics. In each clinic, either the clinic staff or one of the authors (LH) approached all patients who could expect at least half an hour waiting time. Participation was optional and anonymous. If the patients could not complete the questionnaire in time, they were asked to complete it after the consultation. No age restrictions applied; for children, the accompanying parent or guardian answered the questionnaire. If desired or needed (e.g. vision impairment), the questions were read to the patients and the answers written for them (by staff or LH). Patients whose physical or mental status made participation impossible were excluded.

The questionnaire had been newly developed in English in cooperation with epidemiologists, medical doctors, and Kampo specialists. A native speaker and LH translated it to Japanese; it was pretested and revised before it was applied. Its items included the following topics: demographics; medical history including serious illnesses in the last five years, current chronic, and any mental illnesses; diseases or symptoms that had initiated the consultation; some background to the current consultation; the patients’ general preventive strategies; generally preferred therapies (Kampo, Western medicine, both combined). The previous use of Kampo (yes/no) defined the users/non-users subgroups; users were asked for origin/prescriber and disease/symptom (free text). Patients were also asked for their estimation what the most useful treatment would be (Western medicine, Kampo, Western medicine + Kampo, don’t know) for a list of 29 frequent diagnoses or symptoms. The list was based mostly on the works of Furnham [30], but modified for Japanese prevalence (16 diagnoses were left unchanged, 11 deleted from the list or subsumed under broader categories, 14 were added). Further items included the patients’ experiences with Kampo, and other opinions (free-text comments). Also recorded was the degree of agreement (5-point Likert item, 1 = “I don’t agree at all”, 5 = “I totally agree”) on general statements that covered the topics: health and health-related everyday behaviour; Western medicine and Kampo (perceived efficacy, satisfaction, estimation of side effects, philosophical background of Kampo); agreement on reasons for current use or non-use of Kampo. A self-rating of knowledge about Kampo diagnoses and treatment was recorded with a 5-point Likert item (1 = “No knowledge”, 5 = “Full knowledge”).

**Figure 1:**
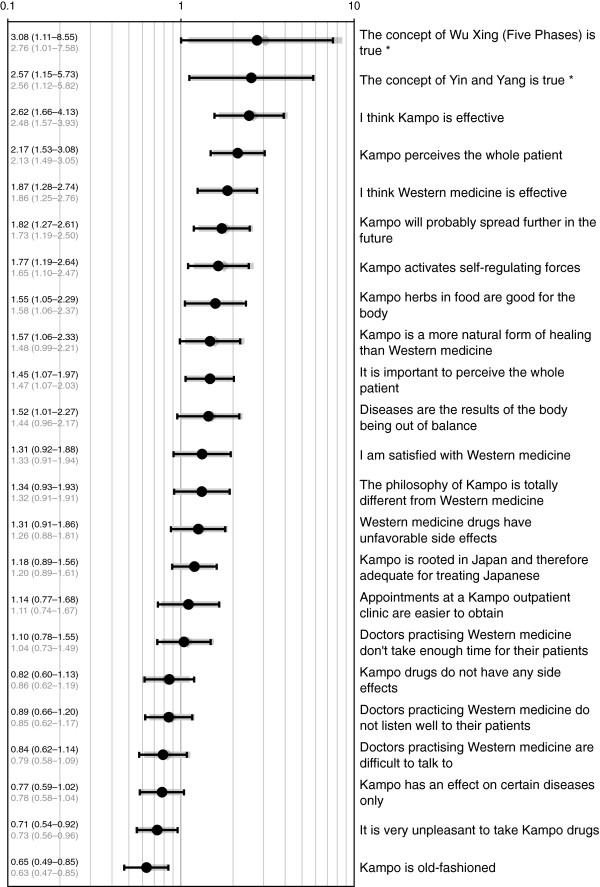
**Opinions as predictors for Kampo use.** Opinions on Kampo and Western medicine as predictors for Kampo use in a logistic regression model. Odds ratios with 95% confidence interval, logarithmic scale. Black: adjusted for chronic disease (see text), grey: unadjusted. * Only patients who knew about concept, broad CI due to low number. “Western medicine” = “conventional” Western biomedicine.

For the total patient population, as well as for the subgroups from each clinic, and the Kampo users and non-users, we calculated descriptive statistics that included absolute and relative frequencies, and for parametric variables the mean and standard deviation (SD). Free text answers were categorized. We used for test statistics non-parametric tests (Mann–Whitney U, Kruskall-Wallis), and for discrete variables the Chi-square test. A first logistic regression analysis was performed to determine socio-demographic and medical aspects that predicted Kampo use (yes/no). The predictors used in this analysis included “type of clinic”, “age”, “gender” (female/male), “married” (yes/no), “education” (high/low), “chronic disease” (yes/no), “mental illness” (yes/no) and “severe disease” (yes/no). The latter responses of categorical variables were used as references, respectively. The Wald test was used to test the statistical significance of each predictor in the model. In the model selection process, backward elimination was used for identifying the best predictors for Kampo use. The goodness-of-fit of the models was assessed based on a log-likelihood ratio test. The significant predictor of the selected model was included as an adjustment variable in several logistic regression analyses to determine the impact of attitudes or opinions about Kampo and Western medicine on predicting the use of Kampo. In each model a different predictor for Kampo use, based on an attitude or opinion about Kampo and Western medicine, was added (cf. Figure 1). The odds ratios (OR) and 95% confidence intervals (CI) were calculated for both models. Missing data was left blank. Calculations were done with PASW Statistics 18 (all ® SPSS Inc.), and IBM SPSS Statistics 21 (® IBM Corp.). As an anonymous survey, our study did not require approval of an ethics committee.

## Results

**Figure 2:**
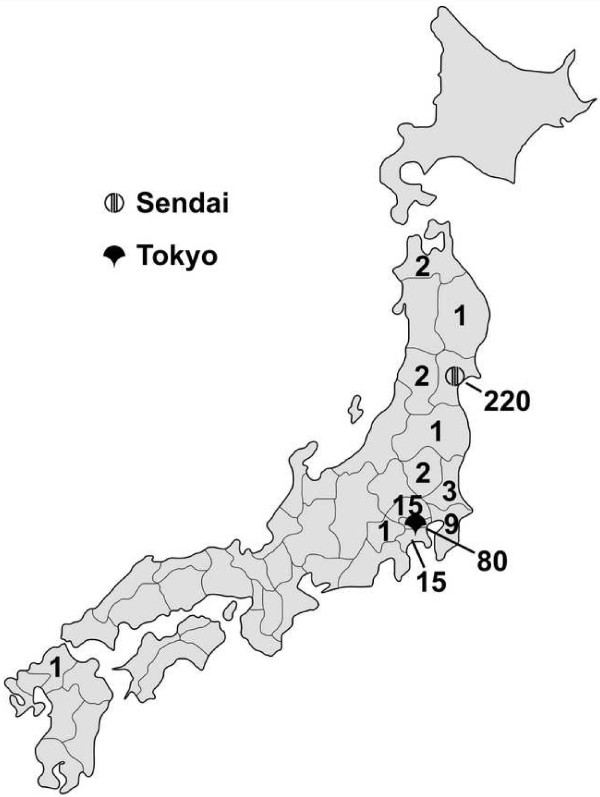
**Study centres and patient residences.** Number of patients from each prefecture. Map based on public domain material [31].

**Figure 3:**
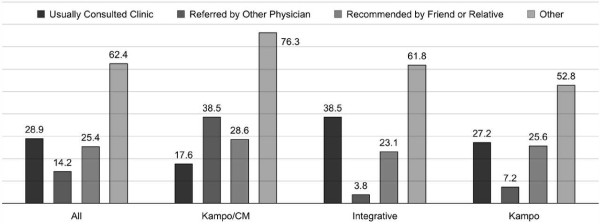
**Reason for choosing study clinic.** Percent of valid answers per clinic. CM (traditional) Chinese medicine. Free text answers (multiple answers possible) included media/internet (n = 40), the specific demand for Kampo (38), proximity (20), recommendations from friends or family (2), and other (41).

Of the 1,376 patients approached for the study, 354 (25.7%) participated. Although the sample size in the different clinics was comparable, the percentage of participating patients varied: Kampo/CM clinic 73.2% (93); Integrative clinic 52.1% (136); Kampo clinic 12.7% (125). More than two third of the patients completing the questionnaire were female (Table 1). Almost all patients (98.0%) were at least high school graduates. Figure 2 shows that almost all patients came from the cities of the respective clinics Sendai (62.5% of the study population, n = 220) and Tokyo (22.7%, 80), few came from the adjacent prefectures (12.5%, 44). Why the clinic was chosen is shown in Figure 3.

**Table 1:** Demographics and occupation

**Demographics**	**All**	**Kampo/CM**	**Integrative**	**Kampo**
	**% (n)**	**% (n)**	**% (n)**	**% (n)**
Total *	100.0 (354)	26.3 (93)	38.4 (136)	35.3 (125)
Sex				
Female	68.4 (240)	72.0 (67)	60.0 (81)	74.8 (92)
Male	31.6 (111)	28.0 (26)	40.0 (54)	25.2 (31)
Age (years)				
Mean ± SD	50.97 ± 15.60	57.46 ± 15.92	47.77 ± 14.71	49.62 ± 14.99
Range	5–88	14–88	5–83	22–77
Education (Graduate)				
Junior High School	2.0 (7)	2.2 (2)	3.0 (4)	0.8 (1)
High School	26.7 (93)	39.1 (36)	29.9 (40)	13.9 (17)
Beyond High School ***	71.3 (248)	58.7 (54)	67.2 (90)	85.3 (104)
Marital Status				
Single	24.7 (87)	24.7 (23)	14.8 (20)	35.5 (44)
Married	63.9 (225)	57.0 (53)	78.5 (106)	53.2 (66)
Divorced	4.5 (16)	6.5 (6)	3.7 (5)	4.0 (5)
Widowed	6.8 (24)	11.8 (11)	3.0 (4)	7.3 (9)
Occupational Situation				
Employed	47.1 (160)	34.1 (31)	56.8 (75)	46.2 (54)
Unemployed (Registered)	2.4 (8)	1.1 (1)	3.8 (5)	1.7 (2)
Without Occupation ****	27.6 (94)	31.9 (29)	23.5 (31)	29.1 (34)
Retired	20.6 (70)	30.8 (28)	13.6 (18)	20.5 (24)
Attending School	2.1 (7)	1.1 (1)	2.3 (3)	2.6 (3)
Retired Plus Working	0.3 (1)	1.1 (1)	0.0 (0)	0.0 (0)
Type of Occupation **				
For a Firm	54.7 (98)	46.7 (21)	61.3 (46)	52.5 (31)
For the Government	11.2 (20)	13.3 (6)	13.3 (10)	6.8 (4)
Teaching	8.4 (15)	8.9 (4)	9.3 (7)	6.8 (4)
Freelancer	8.4 (15)	11.1 (5)	4.0 (3)	11.9 (7)
Self-Employed	14.0 (25)	11.1 (5)	10.7 (8)	1.7 (12)
Entrepreneur	3.4 (6)	8.9 (4)	1.3 (1)	0.8 (1)
Type of Employment **				
Regular	68.6 (116)	74.4 (29)	72.7 (56)	58.5 (31)
Part Time/Side Job (arubeito)	24.9 (42)	23.1 (9)	22.1 (17)	30.2 (16)
Interim Staffing	5.3 (9)	0.0 (0)	25.2 (4)	9.4 (5)
Freelance	0.6 (1)	2.6 (1)	0.0 (0)	0.0 (0)
Regular Plus Part Time	0.6 (1)	0.0 (0)	0.0 (0)	1.9 (1)
Professional Duties **				
Management	23.3 (42)	20.9 (9)	28.0 (21)	19.4 (12)
General Office Work	43.3 (78)	51.2 (22)	38.7 (29)	43.5 (27)
Professional Job	23.3 (42)	16.3 (7)	29.3 (22)	21.0 (13)
Other	10.0 (18)	11.6 (5)	4.0 (3)	16.1 (10)

Percent of valid answers, generally of clinic, * of study population, ** of respective group. *** Technical college, junior college/university, graduate school. **** E.g., household. CM (traditional) Chinese medicine.

Overall, 446 medical complaints (diseases or symptoms, 1.26 per patient) were stated as reasons for the current consultation. They had lasted for an average duration of 8.4 ± 8.9 (mean ± SD) years, varying between clinics (Kampo/CM 10.4 ± 10.2; Integrative 6.3 ± 7.7; Kampo 8.8 ± 8.6 years). The most frequent complaints were classified as neurologic or psychiatric (21.2%, n = 75), cardiovascular (14.4%, 51), pain (14.1%, 50), allergic or dermatological (11.9%, 42), and gastroenterologic (11.6%, 41) disorders. Half of the patients were chronically ill (50.6% of valid answers, n = 178). They actually suffered from a total of 232 ailments, most frequently from cardiovascular (11.3%, 40), neurological or psychiatrical (8.2%, 29) or allergic/dermatological (7.9%, 28) diseases. For the last 5 years before the survey 76 patients reported a total of 88 severe diseases (21.6% of valid answers). The most frequent were gynaecological disorders (4.5%, 16), neurologic or psychiatric disorders (3.1%, 11), and gastroenterological (2.8%, 10) disorders. (Details of diseases and symptoms are available online.)

“Diet” (37.3%, 132) and “Exercise” (33.3%, 118) were the most often practised prevention strategies. As a general treatment preference, most patients wanted the combination of Kampo with Western medicine (54.4%, 179), followed by Western medicine alone (31.3%, 103). Kampo only was preferred by 14.3% (47) of the patients. From the provided list of 29 indications ≥30% of the patients regarded Kampo alone as best treatment option for 7 indications: Hi’e-sho, shoulder stiffness, digestive problems, allergy, stress or anxiety, hay fever, and disturbed sleep. Western medicine only was seen best only for pneumonia. For most indications (n = 25) ≥30% of the patients favoured the combination of Kampo and Western medicine. (Percentages and values per clinic available online.)

Questions on general health-related opinions and behaviour revealed a strong environmental awareness. Patients admitted to taking care for a healthy diet and actively seeking information about health issues (Table 2).

**Table 2:** Health-related opinions and behaviours

**Statement**	**All**	**Kampo/CM**	**Integrative**	**Kampo**
	**Mean ± SD (Median)**	**Mean ± SD (Median)**	**Mean ± SD (Median)**	**Mean ± SD (Median)**
I think global warming is a priority issue	4.05 ± 1.04 (4)	4.12 ± 1.02 (4)	3.93 ± 1.01 (4)	4.13 ± 1.07 (4)
The government should spend more money on improvements to the environment	3.99 ± 0.95 (4)	3.99 ± 0.92 (4)	3.88 ± 0.89 (4)	4.12 ± 1.03 (4)
I try to avoid getting ill	3.96 ± 0.98 (4)	3.91 ± 1.05 (4)	3.82 ± 0.92 (4)	4.15 ± 0.97 (4)
I keep an eye on the nutrition balance of my food	3.95 ± 1.01 (4)	4.10 ± 1.00 (4)	3.67 ± 1.04 (4)	4.14 ± 0.91 (4)
I adjust my diet to suit my physical condition	3.87 ± 0.98 (4)	3.89 ± 0.97 (4)	3.67 ± 0.95 (4)	4.06 ± 0.97 (4)
I read a lot about health in newspapers, books, magazines, etc.	3.79 ± 1.06 (4)	3.75 ± 1.15 (4)	3.66 ± 0.99 (4)	3.96 ± 1.06 (4)
I pay attention to health information on TV and radio	3.68 ± 1.05 (4)	3.76 ± 1.05 (4)	3.44 ± 1.00 (3)	3.88 ± 1.06 (4)
I don’t eat food containing additives and preservatives	3.62 ± 1.09 (4)	3.70 ± 1.11 (4)	3.39 ± 1.03 (3)	3.82 ± 1.09 (4)
I think one should not take synthetic drugs for an extended period of time	3.62 ± 1.06 (3)	3.58 ± 1.10 (3)	3.42 ± 0.99 (3)	3.87 ± 1.08 (4)
I ask my doctor or pharmacist about the drugs prescribed to me	3.60 ± 1.06 (4)	3.65 ± 1.01 (4)	3.44 ± 0.97 (3)	3.75 ± 1.16 (4)
I ask my doctor or pharmacist about the side effects of the drugs prescribed to me	3.50 ± 1.06 (4)	3.53 ± 1.10 (4)	3.25 ± 0.95 (3)	3.76 ± 1.09 (4)
I buy organic food (organically produced vegetables, etc.)	3.21 ± 1.18 (3)	3.29 ± 1.13 (3)	2.98 ± 1.15 (3)	3.41 ± 1.20 (3)
I exclude animal products from my food	2.77 ± 1.12 (3)	2.82 ± 1.05 (3)	2.64 ± 1.06 (3)	2.88 ± 1.22 (3)
When I see an ad about Kampo medicine I don’t know, I ask my doctor about it	2.44 ± 1.18 (2)	2.71 ± 1.16 (3)	2.18 ± 1.12 (2)	2.52 ± 1.20 (2)
I pay attention to the lucky and unlucky days of the Buddhist calendar	2.28 ± 1.22 (2)	2.21 ± 1.10 (2)	2.40 ± 1.23 (2)	2.19 ± 1.29 (2)

Answers on 5 point Likert item, 1 = “I fully disagree”, 5 = “I fully agree”. SD standard deviation. CM (traditional) Chinese medicine. “Western medicine” = “conventional” Western biomedicine.

**Figure 4:**
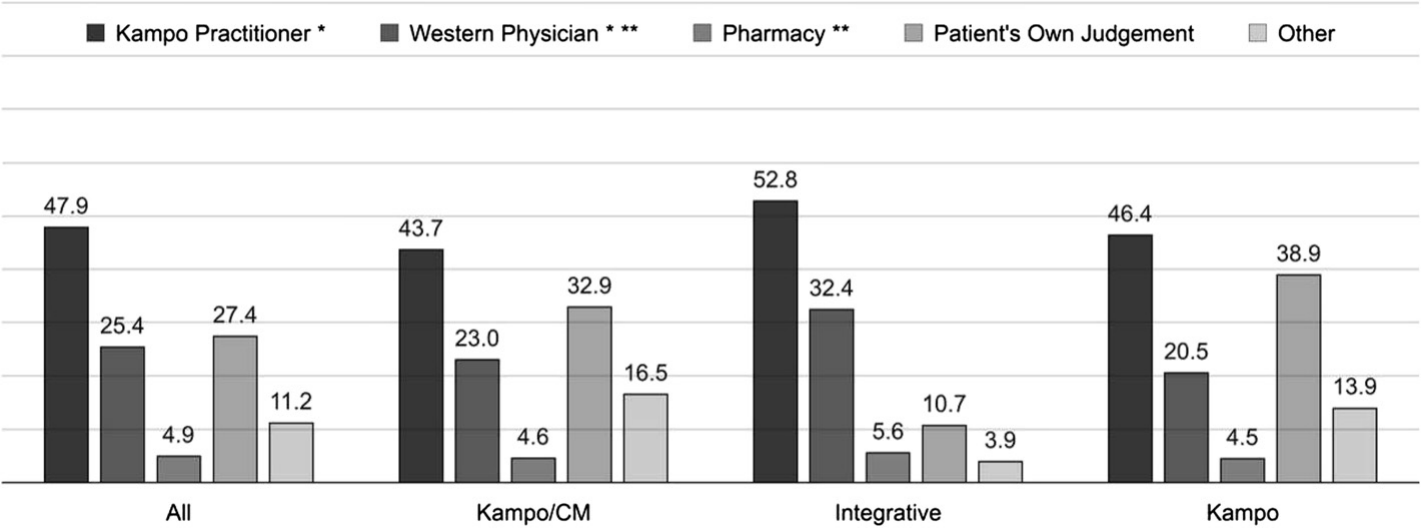
**Who selected the Kampo medication in previous use.** Multiple answers possible. Percent of valid answers per clinic. * Prescription, ** Recommendation. CM (traditional) Chinese medicine.

Of all participating patients, 73.2% (202) were currently using Kampo (Kampo/CM clinic 79.8% (67); Integrative clinic 56.3% (49); Kampo clinic 81.9% (86)). Most patients (83.9%, 297) had used Kampo before (Kampo/CM, 91.5% (85); Kampo 88.0% (110); Integrative 75.0% (102)), the last time 4.0 ± 5.1 (mean ± SD) years ago (Kampo/CM, 2.8 ± 2.9; Integrative 3.2 ± 5.0; Kampo 7.3 ± 6.6). Table 3 lists the most frequent medical complaints for which Kampo had been used then. One third (38.6%) of the respective Kampo medication had not been prescribed or recommended by a health care professional (Figure 4). Most (88.8%, 158) of the chronically ill had experience with Kampo.

**Table 3:** Indications for previous use of Kampo medicines

**Treated medical complaint**	**All**	**Kampo/CM**	**Integrative**	**Kampo**
	**% (n)**	**% (n)**	**% (n)**	**% (n)**
Common Cold	36.2 (105)	31.2 (29)	27.9 (38)	30.4 (38)
Gastrointestinal Complaints	30.2 (88)	24.7 (23)	17.6 (24)	32.8 (41)
Hi’e-sho *	29.0 (84)	31.2 (29)	12.5 (17)	30.4 (38)
Stress/Anxiety Disorder	20.7 (60)	19.4 (18)	15.4 (21)	16.8 (21)
Shoulder Stiffness	20.3 (59)	25.8 (24)	9.6 (13)	17.6 (22)
Gynaecological Disease	18.2 (53)	14.0 (13)	13.2 (18)	17.6 (22)
Allergy	16.8 (49)	11.8 (11)	7.4 (16)	17.6 (22)
Hay Fever	14.8 (43)	16.1 (15)	8.1 (11)	13.6 (17)
Headache/Migraine	13.1 (38)	17.2 (16)	8.8 (12)	8.0 (10)
Disturbed Sleep	12.0 (35)	10.8 (10)	8.8 (12)	10.4 (13)
Low Back Pain	11.0 (32)	12.9 (12)	2.9 (4)	12.8 (16)
Dermatological Disease	9.7 (28)	7.5 (7)	0.7 (1)	16.0 (20)
Bronchitis	6.9 (20)	4.3 (4)	5.9 (8)	6.4 (8)
Asthma	6.5 (19)	8.6 (8)	3.7 (5)	4.8 (6)
Cystitis	6.2 (18)	6.5 (6)	3.7 (5)	5.6 (7)
Muscular Disease	6.2 (18)	8.6 (8)	2.9 (4)	4.8 (6)
Neuralgia	5.5 (16)	7.5 (7)	2.9 (4)	4.0 (5)
Anaemia	4.5 (13)	5.4 (5)	4.4 (6)	1.6 (2)
Depression	4.5 (13)	2.2 (2)	4.4 (6)	4.0 (5)
Hypertension	3.8 (11)	3.2 (3)	3.7 (5)	2.4 (3)
Kidney Disease	3.4 (10)	3.2 (3)	0.7 (1)	4.8 (6)
Hypotension	3.1 (9)	3.2 (3)	1.5 (2)	3.2 (4)
Obesity	3.1 (9)	2.2 (2)	2.9 (4)	2.4 (3)
Rheumatic Disease	2.7 (8)	3.2 (3)	2.2 (3)	1.6 (2)
Cancer	1.7 (5)	1.1 (1)	0.0 (0)	3.2 (4)
Diabetes	1.7 (5)	1.1 (1)	0.7 (1)	2.4 (3)
Gastric Ulcer	1.7 (5)	1.1 (1)	0.7 (1)	2.4 (3)
Pneumonia	1.7 (5)	2.2 (2)	0.7 (1)	1.6 (2)
Addiction (Alcohol, Drugs, Other)	0.3 (1)	1.1 (1)	0.0 (0)	0.0 (0)

Ailments for which Kampo had been used in the past. Percent of valid answers of Kampo users. CM (traditional) Chinese medicine. * Oversensitivity to cold syndrome, see text.

The current use or non-use of Kampo was strongly based on the actual need and its attributed holistic and natural qualities (Table 4). It was seen as more effective and causing fewer side effects than Western treatment. Patients who did not use Kampo currently were open for future use (Table 4).

**Table 4:** Reasons for current Kampo use or non-use

**Reasons**	**All**	**Kampo/CM**	**Integrative**	**Kampo**
	**Mean ± SD (Median)**	**Mean ± SD (Median)**	**Mean ± SD (Median)**	**Mean ± SD (Median)**
For Using Kampo				
Because I believe that Kampo is more holistic	4.15 ± 0.89 (4)	4.27 ± 0.91 (5)	3.56 ± 0.77 (3)	4.43 ± 0.77 (5)
Because Kampo treatment is more natural	4.08 ± 0.90 (4)	4.14 ± 0.97 (4)	3.77 ± 0.82 (4)	4.23 ± 0.86 (4)
Because my doctor suggested it to me	3.86 ± 1.43 (4)	4.08 ± 1.29 (5)	4.31 ± 1.06 (5)	3.38 ± 1.62 (4)
Because Kampo is more effective for my problem than Western medicine	3.88 ± 0.96 (4)	4.10 ± 0.91 (4)	3.49 ± 0.88 (3)	3.97 ± 0.98 (4)
Because Kampo has fewer side effects	3.84 ± 0.99 (4)	3.86 ± 1.09 (4)	3.80 ± 0.96 (4)	3.86 ± 0.96 (4)
Kampo offers a better explanation for my disease than Western medicine	3.58 ± 1.46 (3)	3.87 ± 1.14 (4)	2.94 ± 1.03 (3)	3.80 ± 0.99 (4)
Because I am disappointed with Western medicine and want to try something else	3.05 ± 1.46 (3)	3.12 ± 1.53 (3)	2.37 ± 1.30 (2)	3.44 ± 1.37 (4)
Because there is no treatment in Western medicine for my particular disease	2.93 ± 1.41 (3)	3.12 ± 1.41 (3)	2.30 ± 1.07 (3)	3.23 ± 1.48 (3)
Because someone apart from my doctor suggested it (family, friend)	2.63 ± 1.59 (2)	2.50 ± 1.84 (2)	2.52 ± 1.33 (3)	2.79 ± 1.55 (3)
Because the Western treatment I have received before was distressing	2.61 ± 1.35 (3)	2.60 ± 1.27 (3)	2.36 ± 1.32 (2)	2.78 ± 1.41 (3)
Because treating my illness with Kampo is less expensive than Western medicine	2.40 ± 1.04 (3)	2.43 ± 1.02 (3)	2.68 ± 0.93 (3)	2.19 ± 1.08 (2)
For Not Using Kampo				
I do not need it right now but would use it in the future if necessary	4.02 ± 1.01 (4)	3.78 ± 1.39 (4)	4.02 ± 0.94 (4)	4.50 ± 1.00 (5)
Kampo is too expensive	2.94 ± 1.07 (3)	2.75 ± 1.04 (3)	2.96 ± 1.05 (3)	3.00 ± 1.63 (3)
Taking Kampo medication is inconvenient	2.76 ± 1.19 (3)	2.00 ± 1.31 (1.5)	2.92 ± 1.09 (3)	2.40 ± 1.67 (2)
Western medicine is very effective so that I do not need Kampo	2.37 ± 1.11 (3)	1.63 ± 0.92 (1)	2.56 ± 1.09 (3)	1.60 ± 0.89 (1)
I think Kampo is not effective	2.22 ± 1.27 (2)	2.67 ± 2.55 (2)	2.22 ± 0.92 (2)	1.40 ± 0.89 (1)
I was disappointed when using Kampo before	1.87 ± 0.99 (1.5)	1.63 ± 1.19 (1)	1.94 ± 0.98 (2)	1.50 ± 1.00 (1)

Answers on 5 point Likert item, 1 = “I fully disagree”, 5 = “I fully agree”, SD standard deviation. CM (traditional) Chinese medicine, “Western medicine” = “conventional” Western biomedicine.

The opinions about Kampo and Western medicine valued it as a holistic, body harmonizing treatment, and as a stimulant for self-healing that was considered more natural than Western medicine but similarly effective. Those who knew the philosophical concepts of Yin-Yang and Wu Xing (Five Phases) saw them as valid. Generally, patients saw Kampo more suitable for the Japanese. Western medicine was seen as efficacious, but its physicians were seen to have less time, listen less well and were more difficult to consult. (Statements as in Figure 1, agreement data available online.)

The predictor that best described Kampo use selected by backward elimination was found to be “chronic diseases” (OR 2.88 [1.48-5.58], p = 0.002). Age, severe diseases, type of clinic, education, mental illness, marital status, and gender could be excluded as predictors from the final model due to their minimal contribution to the model outcome. In short, patients who had used Kampo before and were treated in a Kampo-affine clinic suffered more often from chronic diseases than the non-users. Therefore, the presence of “chronic diseases” was used as the only adjustment variable to investigate the influence of opinions on Kampo and Western medicine for Kampo use. The resulting predictors for Kampo use (Figure 1) showed a positive association of Kampo use with beliefs in the underlying philosophy (Wu Xing (adjusted OR 3.08, [1.11-8.55]), Ying and Yang (OR 2.57 [1.15-5.73]), a holistic way of seeing the patient (OR 2.17 [1.53-3.08]), and in Kampo efficacy (OR 2.62 [1.66-4.13]. Interestingly, conviction of the efficacy of Western medicine (OR 1.87 [1.28-2.74]) also predicted for Kampo use.

**Figure 5:**
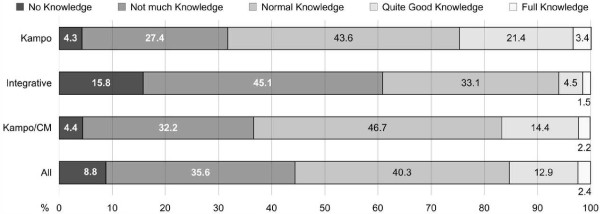
**Kampo knowledge.** Patients’ self-rating of their knowledge. Percent of valid answers per clinic. CM (traditional) Chinese medicine.

Overall, the patients’ self-rated knowledge about Kampo can be described as moderate (Figure 5). The means ± SD ranged between clinics from 2.31 ± 0.85 (Integrative clinic) to 2.92 ± 0.89 (Kampo clinic), i.e., between 2 = “not much knowledge” and 3 = “normal knowledge”.

## Discussion

In our survey at three settings that were typical for Japanese health care and offering Kampo, the responding patients were predominantly female and of somewhat above-average education. Most had used Kampo before. Their opinions were informed by convictions that could be described as “green” or “alternative”, by a sense of responsibility for one’s own health, and by the combination of philosophical concepts of Kampo with a pragmatic approach towards the choice of treatment (Western, Kampo, or both). The use of Kampo was positively associated with the presence of chronic diseases, the attribution of efficacy for both Kampo and Western treatments, the conviction of its natural or holistic qualities, and for some patients, the belief in specific Asian philosophies.

Among the strengths of the study are the diversity of the three clinics (representing different situations and intensities of Kampo usage, and thus, patient populations), the sample size, the inclusion of patients from all socio-economic classes, and the modification of questionnaires from prior studies [30,32] to better accommodate cultural aspects. Balancing the level of detail with feasibility we decided for a 5-point scale. Free text fields allowed getting more details, which in the highly normative Japanese culture constituted an enormous advantage. The included clinics represent typical settings of Japanese routine care that (including Kampo) is mostly provided by outpatient departments (gairai, 外来). An increasing number of universities offer Kampo [33], and patients prefer larger and more prestigious clinics [34]. We had therefore searched for larger outpatient clinics with a focus on Kampo that were also easily accessible for the general population, and, if possible, university clinics. The inclusion of a doctor in his own practice also covered another frequent setting.

The limitations include a possible recall bias, and a low response rate at one site (Kampo clinic). It certainly increased the response rate that clinic staff or one of the authors (LH) approached eligible patients personally. This was not possible in the Kampo clinic, where the small size of the waiting room, too, may have lowered the return rate. Results are certainly influenced by different clinic settings; for example, the long waiting period in the Kampo/Chinese medicine (Kampo/CM) clinic may have selected a different type of patients than the Integrative clinic with its shorter waiting time. A further setting-induced limitation is the exclusion of healthy citizens. They might have differed in their opinions, but would probably have been less interested in medical issues. We also did not expect our population to use Kampo for preventative purposes. Our study cannot claim representativity for Japanese health care; the more rural areas in particular were not represented. Disease prevalence and Kampo use are also likely to vary with the marked climatic change during the year, and the climatic differences across Japan are large but very small between Sendai and Tokyo [35]. The limitations may explain the difference between our results (83.9% of patients had used Kampo before) and general population-based surveys, where a lifetime utilization of 37.5% [5] of Kampo, or a 12-months prevalence of prescription Kampo of 10.0% [3] have been observed.

The majority of participating patients in the clinics offering Kampo were female, similar to another study at a Kampo clinic [21] including 68.4% women. However, in a population-based survey [3], no significant gender predominance was found in the subgroup of Kampo using patients. Almost all patients in our survey had completed high school, in comparison to the Japanese census of 2000 (24.6%) [36]. Costs were unlikely to have caused the difference, because prescription Kampo is almost fully reimbursed within the Japanese health insurance system. One explanation might be the metropolitan sample of the survey. The occupational situation was comparable to the general population [37] with the exception of a lower unemployment rate of 2.4% we observed (compared to about 4% in the survey period) [38]. This might result from our older, and thus for a greater part retired sample group, that in turn is caused by our inclusion of only diseased persons. Kampo is almost solely used in Japan, but our study showed results similar to international research on CAM in general: The patients were predominantly middle-aged, female, and higher educated [39,40] and used CAM in addition to Western medicine [41].

Although Kampo with its holistic philosophy theoretically offers treatments for most medical complaints we found that it was used only for a limited spectrum of diseases. Recommendations for its sole use were only for medical complaints of a less serious nature for which Western medicine sometimes does not offer concepts or satisfying treatments. That diseases and symptoms varied between clinics might be due to demographic variations or clinic specializations. The results from our “Kampo” clinic differed from the results of an earlier survey at the same clinic, when most frequently diseases had been categorized as gynaecological, (23.2%), dermatological, (17.1%), and gastroenterological (15.2%) [19]; the reason for the difference is unclear. Generally, we found that Kampo is used to a lesser extent for muscular diseases. Tradition may have informed public opinion in such a way that for musculo-skeletal problems acupuncture or osteopathy are regarded as best, and Kampo for internistic diseases. The “Hi’e-sho” (commonly 冷え性, experts prefer 冷え症) listed under the diagnoses or symptoms is an “oversensitivity to cold (syndrome)” quite common in Japan [42].

Not every patient was currently taking Kampo. Especially in the Kampo clinic, a high rate of new patients who were yet to receive their prescription answered the questionnaire. Generally, the current Kampo use may have been reduced by available treatment alternatives, especially in the Kampo/CM clinic where patients were also treated with acupuncture and/or moxibustion (without receiving Kampo), and in the Integrative clinic where even more alternatives existed.

In our study, a few less patients (27%) had previously taken Kampo on their own decision (i.e., OTC) than in a survey from 1993 (33%) [21]. Most had used it then after a recommendation by friends (42%), some followed a physician’s recommendation (16%), and 6% of the 198 patients because of media reports (TV, newspaper) [21]. The differences are likely due to Kampo becoming integrated into the medical mainstream (shifting weight from OTC to prescription), to changes in the media landscape (the Internet is likely to have caused much of the difference), as well as to differences in study centres, questionnaires and data acquisition. A study in 2002 [4] found that 19% of patients at a Tokyo general outpatient clinic had used Kampo in the last 12 months.

The stated reasons (musculo-skeletal problems, 15%; gynaecological problems, 13%; less or no side effects, 11%) differed again from our study, likely because of the different questionnaires used. Less perceived side effects was also a reason for using Kampo in 1992 [22]. Disappointment with Western medicine as such was not stated in 1980 [13] and also not seen as a reason by our patients (Kampo users even rated Western medicine more effective than non-users did). The East Asian worldviews that were found to be associated with its use in 1980 [13] seem to have prevailed. So did the notion that Kampo was more a complement to and not a mutually exclusive alternative to Western medicine [13]. We saw it, for example, in the high preference for the combination treatment. It is also reflected in the most frequently stated reasons for the use of CAM in general in Japan as found by others (Kampo being the most wanted, by 69% of the participating patients): treatment under physician’s guidance, 55%; coverage by health insurance, 49%; Western medicine and CAM in same hospital, 47%; higher effect from combining Western medicine and CAM, 43%; multiple approaches, 42% [5]. As seen in 1980, [13] we found that Kampo is used among all social strata and for a broad range of diseases.

Why a higher estimation of effectiveness for both types of medicines predicted for Kampo use is unclear. Perhaps a stronger sense of control over one’s health (as seen in the opinion and prevention parts of the answers) correlates with the openness for treatment alternatives. The opinions on Kampo and the reasons for its use pointed to an inclination towards a certain degree of a “green” or “alternative” worldview. The perceived qualities of Kampo are likely to come from two sources. Satisfying experiences in the past certainly informed the current treatment recommendations, especially when conditions improved that did not get better under Western treatment (or even worsened), as found in a recent study [43]. Secondly, patients’ identification with their choice of treatment [44] may play a not so small role. The moderate knowledge about Kampo and the emphasis on effectiveness suggests that choosing Kampo is less a matter of strong adherence to a theory or worldview than of pragmatism. This is consistent with the wide use and the large number of recommendations for a combination of Kampo and Western medicine. Together with the other results, these show that the investigated population perceives prescribed Kampo as an integrative part of Japanese health care, with particular strengths and limitations.

## Conclusions

Most patients visiting a clinic that provided Kampo had previous experience with Kampo. Behaviour and opinions revealed a mindset with environmental awareness and philosophical reasoning, and a feeling of responsibility for one’s own health. Kampo was used mostly for less serious diseases, and was seen by our population as an integrative part of the health care. Usage was associated with beliefs in philosophical Kampo concepts and its efficacy.

## Competing interests

The authors declare that they have no competing interests.

## Authors’ contributions

CMW and LH developed the study idea; CMW supervised the study and data interpretation. LH, CMW, KW, and TS developed the questionnaire and decided on the setting for the survey. LH was responsible for data acquisition, entry, and analyses; JO calculated the regression analysis and supervised LH data analyses. CMW, LH, KW, TS, JO and TW interpreted the data. LH, TW, CMW drafted the manuscript; all authors revised the manuscript. All authors read and approved the final manuscript.

## Pre-publication history

The pre-publication history for this paper can be accessed here:

http://www.biomedcentral.com/1472-6882/13/108/prepub
